# Relationship Between the Oral Health-Related Quality of Life (OHRQoL) of Mothers and That of Their Adolescent Children: A Retrospective Pilot Study

**DOI:** 10.3390/children12121691

**Published:** 2025-12-13

**Authors:** Adrián Curto, Beatriz Egido, Cristina Gómez-Polo, Kelly Valeria Restrepo-Colorado, Daniele Garcovich, Virginia Franco-Varas, Javier Flores-Fraile

**Affiliations:** 1 Department of Surgery, University of Salamanca, 37007 Salamanca, Spain; begidog24@usal.es (B.E.); crisgodent@usal.es (C.G.-P.); j.flores@usal.es (J.F.-F.); 2 Department of Dentistry, Camilo José Cela University, 28692 Villanueva de la Cañada, Spain; kellyvaleria.restrepo@ucjc.edu; 3 Department of Dentistry, European University of Valencia, 46010 Valencia, Spain; daniele.garcovich@universidadeuropea.es; 4 Faculty of Medicine and Nursing, University of the Basque Country, 48940 Leioa, Spain; virginia.franco@ehu.eus

**Keywords:** adolescents, associated factors, child impact, family impact, mother–child, oral health-related quality of life

## Abstract

**Background:** Oral health-related quality of life (OHRQoL) in children is an issue of growing interest in the scientific literature, with various studies focused on the adolescent population published in recent years. Nevertheless, few studies have evaluated the possible influence of the OHRQoL of mothers on that of their adolescent children. The aim of this study was to analyse the correlation between the OHRQoL of mothers and that of their adolescent children at their first oral examination. **Methods:** This retrospective pilot study was performed at the Dental Clinic of the University of Salamanca (Spain) between 2023 and 2025. The OHRQoL of 130 adolescent patients (from 11 to 14 years old) who visited the dentist for the first time was analysed. The mothers of these children were also interviewed to evaluate their OHRQoL; the adolescent patients completed the Spanish version of the Child Perceptions Questionnaire (CPQ-Esp_11–14_) before their first dental consultation, and their mothers completed the Oral Health Impact Profile-14 (OHIP-14). **Results:** The study population consisted of 130 mothers (mean age: 43.0 ± 2.66 years) and their respective adolescent children (mean age: 12.5 ± 1.3 years) (65 boys and 65 girls). Among the adolescent patients, the highest score was obtained for the social well-being dimension of the CPQ-Esp_11–14_ (25.01 ± 4.10), whereas the lowest was obtained for the oral symptoms dimension (10.88 ± 3.78). A higher score on the physical pain dimension of the OHRQoL for the mothers was related (*p* < 0.01) to higher scores on the emotional and social well-being dimensions of the CPQ-Esp_11–14_ for their children. **Conclusions:** Considering the limitations of this study, it can be concluded that the OHRQoL of mothers has some effect on that of their adolescent children when visiting a dentist for the first time.

## 1. Introduction

The quality of life (QoL) of a person is defined as the set of conditions that contribute to their social and personal well-being. QoL is important for evaluating people’s physical and mental health, as well as their oral health. The concept of oral health involves the ability to speak, chew, smile and express emotions confidently using facial expressions that do not show signs of pain or discomfort [1,2,3]. The term oral health-related quality of life (OHRQoL) is used in this context. Among other things, OHRQoL describes a person’s comfort when eating, sleeping or having social encounters, and it also involves an individual’s self-esteem and satisfaction with respect to their oral health [3]. OHRQoL provides information about the impact that oral conditions have on day-to-day activities from a patient’s point of view [4]. Evaluating the OHRQoL of patients can help dentists to identify the concerns described by patients and prioritise different options for dental treatment [5]. The OHRQoL of minors and their parents’/guardians’ perceptions of it can be influenced by different factors, such as age, sex, environmental factors, sociodemographic factors and oral conditions [6,7,8,9,10,11]. OHRQoL also quantifies the impact that facial aesthetics have on young patients [12]. Different questionnaires have been developed to quantify OHRQoL for both adult and paediatric patients. Many questionnaires have been validated in different regions or languages [3,13]. Among these questionnaires are the Child Perception Questionnaire (CPQ), the Child-Oral Impact Daily Performance (Child-OIDP), and the Early Childhood Oral Health Impact Scale (ECOHIS) [6,14,15,16].

Numerous studies in the scientific literature have evaluated OHRQoL in paediatric and adolescent patients [1,17,18,19,20]. However, few studies have analysed the correlation between the OHRQoL of parents and its possible influence on that of their adolescent children [21,22,23]. For example, some studies have reported a significant correlation between good OHRQoL among parents and a lower incidence of dental cavities among their children [24,25]. It has been concluded that parents or guardians of paediatric patients play a fundamental role in the prevention and management of oral health problems in their children. According to the World Health Organization, the stages of preadolescence and adolescence are transition periods that influence the health of individuals and their predisposition to disease during adulthood [26]. Previous studies that have evaluated this correlation have not focused on evaluating the OHRQoL of adolescent patients at their first visit to a dentist. Assessing the OHRQoL of patients—in this case, adolescent patients—before their first dental visit is important so that the professional can understand the impact and perception that adolescents have of their oral health, and its implications in their social environment.

The null hypothesis in this study was that mothers’ perceptions of their OHRQoL have no relationship with the OHRQoL reported by their adolescent children.

The primary goal of this study was to analyse the possible influence of the OHRQoL of mothers on that of their adolescent children before their first oral examination. The secondary goal was to quantify the OHRQoL of an adolescent population who had never previously undergone an oral examination.

## 2. Materials and Methods

### 2.1. Ethics

This study was approved by the Research Ethics Committee of the University of Salamanca (protocol number 1078 dated 27 November 2023). This study followed the directives established by the Helsinki Declaration for research with humans, as well as the Strengthening the Reporting of Observational studies in Epidemiology (STROBE) guidelines for carrying out observational studies [27]. The participants (both children and their mothers) were informed about the procedures of the study. Before inclusion in the study, informed consent was obtained from both the adolescent patients and their mothers.

### 2.2. Study Design

**Figure 1:**
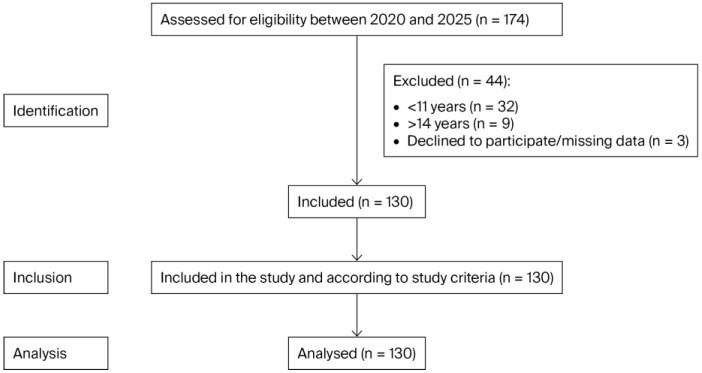
STROBE flow chart.

This cross-sectional observational study was carried out at the Dental Clinic of the University of Salamanca between December 2023 and July 2025. This study analysed the OHRQoL of a sample of 130 mothers and their adolescent children (aged between 11 and 14 years). Previous studies have also evaluated the age range of 11 to 14 years, considering this range as adolescent patients [23,28,29]. The total sample consisted of 130 mothers and 130 adolescents who were chosen consecutively from the patients who visited the Dental Clinic (Figure 1). The adolescent patients were patients who had never undergone a dental examination. Because it was a pilot study, only convenience sampling was performed. Moreover, due to the characteristics of this work (adolescent patients who had not previously attended a dental appointment) and the lack of previous studies, it was not considered necessary to calculate the required sample size in advance.

### 2.3. Inclusion and Exclusion Criteria

The inclusion criteria for adolescent patients were as follows: (i) patients between the ages of 11 and 14 years; (ii) patients undergoing their first dental examination; (iii) patients who could visit the dentist with their mother. The exclusion criteria were as follows: (i) patients with a physical or mental disability; (ii) patients who had received dentofacial orthopaedic or orthodontic treatment; (iii) patients with systemic diseases; (iv) patients under continuous treatment with pharmaceuticals.

### 2.4. Procedures

The OHRQoL of the participants was analysed using the Spanish version of the Child Perceptions Questionnaire validated for a population between 11 and 14 years old (CPQ-Esp_11–14_) [30]. The Child Perceptions Questionnaire 11–14 comprises 37 items and encompasses four dimensions of OHRQoL: oral symptoms, functional limitations, emotional well-being and social well-being. Items are scored on a 5-point Likert scale ranging from 0 to 4 points (0: never; 1: once or twice; 2: sometimes; 3: frequently; and 4: every day or almost every day); a higher score is indicative of worse OHRQoL [31].

The OHRQoL of the mothers was quantified using the Spanish version of the Oral Health Impact Profile-14 (OHIP-14). The OHIP-14 consists of 14 items that analyse the following seven domains of OHRQoL: functional limitations, physical pain, psychological discomfort, physical disability, psychological disability, social disability, and disability. The items are scored using a 5-point Likert scale (0 = never, 1 = almost never, 2 = occasionally, 3 = quite often, and 4 = very often) [32,33].

Both questionnaires (CPQ-Esp_11–14_ and OHIP-14) were given to the patients and their mothers at the same appointment. The OHIP-14 and CPQ-Esp_11–14_ were provided to study participants once before the adolescents’ oral examination. The questionnaires were provided by a single examiner who was a clinical practitioner of paediatric dentistry (A.C.). The adolescents completed the questionnaire prior to their oral examination and were separated from their mothers at the time. The adolescents and their mothers were given a few minutes to complete the questionnaires and were provided information by the examiner to help them.

### 2.5. Statistical Analysis

IBM SPSS Statistics (Version 28.0) was used to analyse the data. Descriptive analysis (mean, standard deviation median, and range) was performed for CPQ-Esp_11–14_ and OHIP-14 total scores and individual items. Spearman’s correlation coefficient (rho) was used to analyse the correlation between the OHRQoL of mothers and that of their adolescent children. A *p* value less than 0.05 was considered to be significant.

## 3. Results

### 3.1. Baseline Characteristics of the Participants

Data were collected from 130 adolescent patients aged between 11 and 14 years who were undergoing their first dental examination. Patients aged 13 years were the most common (*n* = 41; 31.5%), and patients aged 11 years were the least common (*n* = 28; 21.5%). The sex ratio of the participants was 1:1; in other words, the sample was composed of 50% (*n* = 65) male participants and 50% (*n* = 65) female participants. Consequently, no statistically significant differences in relation to age or the participants included in the study were observed. OHRQoL data were also collected from the patients’ mothers. The mothers had a mean age of 43.0 ± 2.66 years, ranging between 39 and 51 years (the median age was 42 years).

### 3.2. Analysis of the OHRQoL of the Adolescent Patients

The OHRQoL of the adolescent participants was evaluated using the CPQ-Esp_11–14_, which comprises four OHRQoL dimensions. The highest mean score was observed for the social well-being dimension (25.01 ± 4.10 points), in contrast to the oral symptoms dimension, which had the lowest mean score (10.88 ± 3.78 points) (Table 1).

Considering the average values (means/medians) of the CPQ-Esp_11–14_ questionnaire, it can be concluded that the OHRQoL of the adolescent population studied falls within the middle range of the scoring scale.

### 3.3. Analysis of the OHRQoL of the Participants’ Mothers

When the OHRQoL of the participants’ mothers was analysed, the physical pain dimension of the OHIP-14 had a higher mean score (5.89 ± 1.40 points) than the disability dimension, which had a lower mean score (4.36 ± 1.52 points). With respect to the descriptive values, in practically all the dimensions of the OHIP-14, the means (means and medians) exceeded the central point (value of 4 points) on the scale of possible response values (0–8 points), which may indicate that the OHRQoL of the mothers tended to be more negative than positive. This was also observed for the total OHIP-14 score, where the mean score (38.05 ± 4.47) exceeded the central point (value of 28 points) on the scale of possible response values (0–56 points) (Table 2).

### 3.4. Correlation Between the OHRQoL of the Mothers and That of Their Children

Upon analysing the relationship between the OHRQoL of the mothers and that of their adolescent children, in general, no sufficiently intense relationships reached statistical significance. In general, there is not sufficient statistical evidence to confirm a relationship between the OHRQoL of mothers and that of their children on the basis of the sample analysed, except two cases. The physical pain dimension (OHIP-14) score of the mothers was correlated with the emotional well-being (CPQ-Esp_11–14_) (r = 0.24; *p* < 0.05) and social well-being (CPQ-Esp_11–14_) (r = 0.23; *p* < 0.05) scores of their children. In other words, the higher a mother’s score on the physical pain dimension, the greater the impact on her child’s scores in emotional and social well-being dimensions (*p* < 0.05) (Table 3).

## 4. Discussion

The goal of this study was to evaluate the relationship between the OHRQoL of mothers and that of their adolescent children. In this study, only a significant relationship between the physical pain dimension score of the mothers (OHIP-14) and the emotional and social well-being dimension scores of the children (CPQ-Esp_11–14_) was observed. In this case, a higher maternal score on the physical pain dimension was related to a greater impact on the aforementioned OHRQoL dimensions of the children. The possible influence of the OHRQoL of the mothers on that of their adolescent children has still not been explored. Various tools have been developed to evaluate the OHRQoL of children. These questionnaires have been validated for different age ranges. The most commonly used questionnaires are the CPQ, the Child-OIDP, the Child Oral Health Impact Profile (COHIP) and the Early Childhood Oral Health Impact Scale (ECOHIS) [14,15,16,34,35]. There are two versions of the CPQ, one for children aged from 8 to 10 years old (CPQ_8–10_) and one for children aged from 11 to 14 years old (CPQ_11–14_) [34,35]. Various questionnaires have also been developed for the adult population to analyse OHRQoL, such as the Oral Health Impact Profile-14 (OHIP-14), the Oral Health Impact Profile-49 (OHIP-49), the General Oral Health Assessment Index (GOHAI), and the Oral Impacts on Daily Performance (OIDP) [36,37,38,39,40]. The Oral Health Impact Profile (OHIP) was used in this study because of its good psychometric properties [38,41]. The original version of the OHIP has 49 questions; because the original version takes longer to complete, in this study, the OHIP-14 was used. This instrument is considered more practical than the OHIP-49 and has also been shown to be a trustworthy tool with good reliability and validity [38,42]. The Spanish version of the CPQ_11–14_ (CPQ-Esp_11–14_) was used for the children in this study, and the OHIP-14 was used for the mothers. Both questionnaires have been widely used in previous studies [32,43,44,45,46,47,48].

Oral conditions affect several aspects of OHRQoL in the paediatric population, such as cavities [6,49], dental trauma [9], molar incisor hypomineralization [50,51], the need for orthodontic treatment [7], bruxism [52] and dental anxiety/pain experienced during dental treatment [10]. All the factors described above can impair the OHRQoL of paediatric patients, as can other sociodemographic factors [53]. One important, widely studied factor is the impact of malocclusion on the OHRQoL of the paediatric population. The severity of a malocclusion is associated with a greater negative impact on the OHRQoL of children [54,55,56,57]. In the preschool population, a relationship between the maternal level of dental anxiety and the OHRQoL of children has been confirmed. It has been concluded that high levels of dental anxiety among mothers influence their children and negatively impact their OHRQoL [58,59].

Yang et al. [21] evaluated the influence of different family factors and of parents’ OHRQoL on the OHRQoL of their children (aged between 3 and 5 years old). They concluded that parental age, the family’s income level, and the family’s place of residence were factors influencing the OHRQoL of their children. They also reported that the OHRQoL of parents (who completed the Oral Health Impact Profile-5 (OHIP-5)) was significantly related to the OHRQoL of their children (who completed the ECOHIS) [21].

In 2022, Velasco et al. [22] analysed the effects of different demographic and socioeconomic conditions of parents on the OHRQoL of their children. The study confirmed that the parents’ degree of knowledge about their children’s oral health status was not associated with an increase in the OHRQoL of their preschool-aged children (between 2 and 4 years old). The presence of cavities and a greater number of siblings negatively impacted the OHRQoL of the preschool population studied [22]. Similar results were also obtained by Sun et al. [23] in 2022. The authors concluded that the level of education of mothers positively influences the OHRQoL of their adolescent children (aged between 12 and 15 years old). The author used the CPQ_11–14_ [23], which was also used in this study. Similar studies, such as that conducted by Costa et al. [60] in 2017, researched the effect of depressive symptoms and anxiety on young mothers (aged 13–19 years old) with respect to the OHRQoL of their children (aged between 2 and 3 years old); this author used the ECOHIS questionnaire. Worse OHRQoL among children was associated with maternal symptoms of anxiety and depression [60].

A recent study by Khattab et al. [61] reported that there is no relationship between oral habits (primarily nail biting) among children (aged between 5 and 7 years old) and their OHRQoL from the point of view of their mothers.

In this study, a meaningful effect of sex on the OHRQoL of the adolescent participants was not observed. A review of the scientific literature revealed that the results of the various published studies are contradictory. The disparity regarding the possible effect on the OHRQoL of paediatric patients may be due to the age ranges of the samples analysed or the sample sizes and the specific inclusion/exclusion criteria of each study [8,62,63,64]. For example, Sun et al. [23], Thiruvenkadam et al. [65] and Kumar et al. [66] reported that boys had worse OHRQoL than girls. Nevertheless, other authors have reported better OHRQoL among boys than girls; this association can be explained by girls’ greater concern for their oral health [11,67,68,69,70]. In contrast to this study, the study by Sun et al. [23] revealed that adolescent males had higher scores on the oral symptoms dimension of the CPQ_11–14_ than adolescent females.

An analysis of the effect of age on the OHRQoL of the adolescent population revealed that there was a significant relationship (*p* < 0.05) only in the oral symptoms dimension of the CPQ-Esp_11–14_. In this dimension, the greater the age, the lower the score; in other words, there was a lower impact on OHRQoL. A significant effect of age was not reported for the remaining CPQ-Esp_11–14_ dimensions.

Interestingly, in this study, a statistically significant correlation (*p* < 0.01) was observed between the physical pain dimension score of the mothers and the emotional and social well-being dimension scores of their children. In other words, higher levels of physical pain among mothers were related to better well-being among their adolescent children, even though, a priori, this is contradictory.

A previous study by the authors of this study aimed to analyse the need for orthodontic treatment in adolescent patients with asthma and how their pathology could influence their OHRQoL. It was concluded that sex, as in this study, did not influence the OHRQoL score according to the CPQ-Esp_11–14_. The greatest score was obtained for the social well-being dimension (15.72 ± 1.91 points), and the lowest for the oral symptoms dimension (7.64 ± 1.39 points). These results are consistent with the results described in the present study. Similar results were also obtained when the relationships between age and the different OHRQoL dimension scores of the adolescents were analysed. In this case, it was observed that, in the oral symptoms and functional limitation dimensions, the greater the age, the lower the score obtained on the OHRQoL questionnaire, the results of which are similar to those obtained in this study [62].

Other studies affirmed that age does not significantly influence the OHRQoL of the paediatric population. It must be specified that this study focused on a very specific age range: the adolescent population aged between 11 and 14 years [8,11,64,71].

As described above, there are very few published studies that have evaluated the correlation between the OHRQoL of parents and that of their adolescent children.

### 4.1. Strengths of This Study

This study has several strengths that deserve to be highlighted. First, this is one of the first studies to evaluate the OHRQoL of adolescent patients at their first visit to a dentist. Notably, a limited number of studies have analysed the relationship between the OHRQoL of mothers and that of their adolescent children. The population analysed was homogeneous with respect to sex and age. Another strength of this study is that research such as that described here is important for analysing adolescent patients because this population group is in a transition period subject to major changes that can be prolonged into adulthood.

It is important to consider the family, specifically the mother, when planning and implementing interventions to promote and improve the dental health and OHRQoL of adolescent patients.

### 4.2. Limitations

The results of this study should be interpreted with caution because of several limitations. One of the limitations of this study was the sample size. As this was a pilot study, a consecutive sample of patients seeking dental care was analysed. Another limitation was that, in this study, patients from a single centre and health institution were analysed. It is possible that there is some effect of the geographic region in which patients reside on their OHRQoL. Multicentre studies and studies analysing changes in the OHRQoL of adolescent patients before and after dental treatment, including an analysis of dental anxiety in patients who are visiting the dentist for the first time and in their parents, should be performed.

## 5. Conclusions

In conclusion, this study provides new information about the impact of the first visit to a dentist on an adolescent population and the relationship between the OHRQoL of mothers and that of their underage children. In summary, considering the limitations of this study, the OHRQoL of mothers may be related to that of adolescent children who have not previously visited a dentist. In the population studied, sex did not influence the OHRQoL of adolescent patients.

## Figures and Tables

**Table 1:** Exploratory and descriptive analysis of the OHRQoL (CPQ-Esp_11–14_) of the adolescent patients (*n* = 130).

CPQ-Esp_11–14_	Mean	SD	Range (Minimum–Maximum)	Median
Oral symptoms	10.88	3.78	5–22	10
Functional limitations	17.58	3.68	10–25	16
Emotional well-being	16.14	2.79	11–21	16
Social well-being	25.01	4.10	19–35	24

SD: Standard deviation.

**Table 2:** Exploratory and descriptive analysis of the OHRQoL (OHIP-14) of the mothers (*n* = 130).

OHIP-14	Mean	SD	Range (Minimum–Maximum)	Median
Functional limitations	5.88	1.40	4–8	6.00
Physical pain	5.89	0.63	5–7	6.00
Psychological discomfort	5.38	0.93	4–7	5.00
Physical disability	5.66	0.63	5–7	6.00
Psychological disability	5.28	1.09	4–8	5.00
Social disability	5.59	0.91	4–7	6.00
Disability	4.36	1.52	2–7	4.00
Total OHIP-14	38.05	4.47	33–49	36.00

SD: Standard deviation.

**Table 3:** Correlations between the OHRQoL (OHIP-14) of the mothers and that of their children (CPQ-Esp_11–14_) (*n* = 260).

Variables	CPQ-Esp_11–14_/ Oral Symptoms	CPQ-Esp_11–14_/ Functional Limitations	CPQ-Esp_11–14_/ Emotional Well-Being	CPQ-Esp_11–14_/ Social Well-Being
	rho (*p* Value)	rho (*p* Value)	rho (*p* Value)	rho (*p* Value)
OHIP-14/Functional limitations	0.11 (0.101)	0.03 (0.360)	0.10 (0.135)	0.12 (0.096)
OHIP-14/Physical pain	0.03 (0.366)	0.08 (0.194)	0.24 (0.003) *	0.23 (0.004) *
OHIP-14/Psychological discomfort	0.05 (0.277)	0.00 (0.495)	0.10 (0.123)	0.10 (0.139)
OHIP-14/Physical disability	0.09 (0.166)	−0.02 (0.402)	0.04 (0.330)	0.09 (0.149)
OHIP-14/Psychological disability	0.05 (0.290)	−0.05 (0.279)	−0.0 (0.296)	−0.07 (0.223)
OHIP-14/Social disability	−0.07 (0.209)	−0.04 (0.313)	0.10 (0.134)	0.04 (0.337)
OHIP-14/Disability	0.06 (0.233)	0.00 (0.492)	−0.13 (0.065)	−0.07 (0.220)
OHIP-14/Total OHIP	0.07 (0.225)	−0.02 (0.419)	0.02 (0.431)	0.02 (0.390)

*: significant (*p* < 0.05).

## Data Availability

The data presented in this study are available upon request from the corresponding author.

## Abbreviations

The following abbreviations are used in this manuscript:

Child-OIDP	Child-Oral Impact Daily Performance
COHIP	Child Oral Health Impact Profile
CPQ	Child Perception Questionnaire
ECOHIS	Early Childhood Oral Health Impact Scale
OHIP-14	Oral Health Impact Profile-14
OHRQoL	Oral health-related quality of life
QoL	Quality of life
SD	Standard deviation
